# The Chemotherapeutic Potentials of Compounds Isolated from the Plant, Marine, Fungus, and Microorganism: Their Mechanism of Action and Prospects

**DOI:** 10.1155/2022/5919453

**Published:** 2022-10-10

**Authors:** Ashok K. Shakya, Rajashri R. Naik

**Affiliations:** ^1^Pharmacological and Diagnostic Research Center, Faculty of Pharmacy, Al-Ahliyya Amman University, Amman 19328, Jordan; ^2^Pharmacological and Diagnostic Research Center, Faculty of Allied Medical Sciences, Al-Ahliyya Amman University, Amman 9328, Jordan

## Abstract

Research on natural products mainly focuses on developing a suitable drug to treat human disease. There has been a sharp increase in the development of drugs from natural products. Most of the drugs that are available are from the terrestrial origin. Marine natural products are less explored. Oceans are considered as a vast ecosystem with a wide variety of living organisms and natural products that are unexplored. Large numbers of antitumor drugs are from natural sources such as plants, marine, and microorganisms. 80% new chemical entities that were launched over the past 60 decades were from a natural source. In this article, the anticancer potential from the natural source such as plants, fungi, microorganisms, marine, and endophytes has been reviewed. Emphasis is given on the compound from the marine, plant, and of bacterial origin. Finally, we consider the future and how we might achieve better sustainability to alleviate human cancer suffering while having fewer side effects, more efficacies, and causing less harm than the present treatments.

## 1. Introduction

During the early era, nature was the only solitary source for the therapeutic agents serving as pharmacy. Natural products (NPs) are derived from the natural source such as plants, animals, fungi, microorganisms, and marine organisms. The products from the natural source also serve as a nutrient, and with potential biological activity, these are often referred to as nutraceuticals. There are a large number of literature studies present on the nutraceuticals, but, here, we are going to emphasize on the chemotherapeutic potentials from the natural sources and some of them that are under the clinical trial for their efficiency as a potential chemotherapeutic agent [1]. The bioactive compounds extracted from natural products have significant medicinal properties [2], and since ancient times, these NPs have played an eminent role in curing human ailments. Indian medicinal system is considered as one of the oldest and organised system of medicine which is mainly influenced by Ayurveda, Siddha, Unani, and Homeopathy, and all these systems rely on natural products derived from plant terrestrial, animals, microorganisms, and marine products to treat various harmful diseases. Natural products since ancient times have played a significant role in preventing various human diseases. Traditional medicines that depend on natural products are gaining popularity in clinical medicine and pharmaceutical research and are widely accepted as conventional therapy to that of the currently available therapeutic options [3, 4]. Many studies are carried out, and these studies focus on the role played by herbs in the management, prevention, and treatment of various diseases because they are cost-effective, more efficient, and are with lesser side effects [5, 6]. Research on natural products mainly focuses on developing a suitable drug to treat human disease, and since 1981 to 2019, a total of ∼1946 small molecules with therapeutic properties have been approved and 65% of the marketed drugs were from natural sources. These natural products were either used as it is unaltered or was modified and developed based on their original structure [7]. In general, natural products have complex structures with well-defined spatial orientation, and these molecules are active pharmaceutical ingredients. In fact, half of the drugs that are discovered and developed so far are based on the structure of the natural product or are from the natural product [7, 8] of which 20 of the best-selling medicines are from or related to natural products. At present, most of the therapeutic products that are from natural source are of terrestrial origin [9].

Over the last 30 years, 61% of anticancer compounds and 49% of the anti-infective drugs that form a substantial market share are derived or developed from a natural source and have been approved. It may be noted that, in 2010, out of 20 molecules launched to treat various disease, 10 of them were directly from NP or developed from NP, and the majority of them were anticancer molecules [10]. From natural sources such as plants, microorganisms, and marine organism, a large number of antitumor drugs have been identified and derived over the past 60 years [11]. There are 236 new chemical entities (NCEs) launched as potential chemotherapeutic agents and 80% of them are from natural source or were developed depending on the structure of the compound from these sources. It may be observed that, over the past three decades from 1980s to 2012, there has been a sharp increase in the development of drugs from a natural sources, and from 2010 to 2012, the number of natural products approved increased [12]. The history of natural products being used as anticancer agents was explored with the discovery of the first anticancer agent from the plant *Podophyllum pelltatum* and the anticancer agent podophyllotoxin **(1)** that arrests cell division by inhibiting the enzyme topoisomerase II [13]. Vincristine (**2**) and vinblastine (**3**) are dimeric alkaloids isolated from the Madagaskar periwinkle plant (*Catharantus roseus*) and exhibit significant cytotoxic activity and are used in the antitumor therapy as antineoplastic agents. Among the entire natural products, the marine natural products (MNPs) have shown higher and most significant bioactivity than any other products [14]. In the present article, we are trying to focus on the anticancer agents that are discovered or developed from natural sources. Here, we are trying to give emphasis on the plant origin and anticancer agent from marine sources and from bacterial origin. In conclusion, we will discuss about the future prospects.

### 1.1. Anticancer Agent from Plant Source

The WHO estimates that, in some of the countries in Asia and Africa, more than 80% of the population depends on traditional medicine for their primary health care [15]. Traditional medicine depends on herbal products and is in great demand. Plants have played a significant role in developing traditional medicine and are gaining popularity. The international market for herbal products is estimated to grow up to 5 trillion dollars by 2050 [16]. Plants contain a large number of molecules with anticancer potentials and around 60% of the anticancer drugs are derived from plant sources either directly or indirectly [17]. Traditional medicine consists of herbal products to treat various ailments and have played a vital role in treating cancer [18]. One of the well-known anticancer agents derived from plant is paclitaxel (**4,** Taxol®) [19] derived from the plant *Taxus brevifolia* Nutt. (western yew). It is now extracted from endophytic fungus like *Taxomyces andreanae* and by other endophytic fungi [20, 21] making it possible for its extraction through microbial fermentation. The name of the compound, its source, and the mechanism of action (of all the compound discussed in the review) are explained briefly in Table 1. The molecular structure of Taxol consists of A, B, and C rings with two hydroxyl, two acetyl, one benzoyl group, and one oxetane ring. The anticancer activity of Taxol is due to the side chain A along with benzoyl group C2 and the oxetane; its anticancer activity is preserved by the C3 amide acyl group in the C12 chain, while the hydroxyl group at C2 increases the activity [43]. Taxol inhibits the disassembly of the microtubules by binding to the polymerized microtubule [44–46]. Microtubule is made up of *α* and *ß* tubulin subunits. Taxol also stabilizes and maintains the dynamics of the tubulin polymer. It also decreases the association of microtubule-associated proteins (MAPs) and binding of Taxol to MAP , by further stabilizing it. These changes prevent the formation and function of mitotic spindle during cell division resulting in inhibition of the cell division and in turn preventing cell proliferation [43, 44, 47, 48]. Another compound docetaxel (**5**) is a semisynthetic compound and exhibits antineoplastic activity.

It took almost 25 years for Taxol since its discovery in 1970s to be marketed for the said purpose. In 1992, FDA approved Taxol for the treatment of the metastatic ovarian cancer. Taxol exhibited emulating results on other types of cancer such as head, neck, lung, and breast cancer [17] (Table 1). Another anticancer agent that was approved for treating cancer is camptothecin **(6)**. It is isolated from *Camptotheca acuminate* bark. Camptothecin is a quinoline alkaloid that inhibits the enzyme topoisomerase and is approved for the use in various countries. The rate of response of drug depends on a large number of factors; one such factor is the type of cancer it is used in the treatment. In case of Taxol, the response rate of ovarian cancer was estimated to be approximately 30%, and in case of metastatic breast cancer, it was estimated to be 56%. This success has led to the increase in the sale of Taxol [49, 50].

Some of the plant-based anticancer agents under clinical trials are flavopiridol—a cyclin-dependent kinase inhibitor [51]. It is isolated from *Amoora rohituka* (Andersonia) stems and leaves, is an synthetic alkaloid, and was later isolated from *Dysoxylum binectaiferum* (Maliaceae) (Table 2) [64]. Flavopiridol interferes with the phosphorylation of the cyclin-dependent kinases inhibiting their activation that results in blocking the progression at gap 1 (G1) or gap 2 (G2) phase of the cell cycle. In phase I, clinical trial flavopiridol (**7**) showed dose-dependent toxicity like secretory diarrhea [65], but response in the success rate on different types of solid and hematological malignancies has led to the initiation of phase II clinical trials. In phase II clinical trials, patients with colorectal, prostrate, renal cell, small cell lung carcinoma, and also on non-Hodgkin's lymphoma and chronic lymphocytic leukemia were tested as presented in Table 2. All the chemical compounds isolated from biological sources that are under clinical trials are presented in Table 2.

At present, a large number of compounds derived from plants are being investigated for their potential anticancer activity; for instance, homoharrintonine, is an alkaloid extracted from the plant *Cephalotaxus harringtonia* (Cephalotaxacea) grown in China. It exhibited significant efficiency against various leukemia [66]. Homoharrintonine inhibits the synthesis of protein, and this results in blocking the progression of the cell cycle [67].

**Figure 1:**
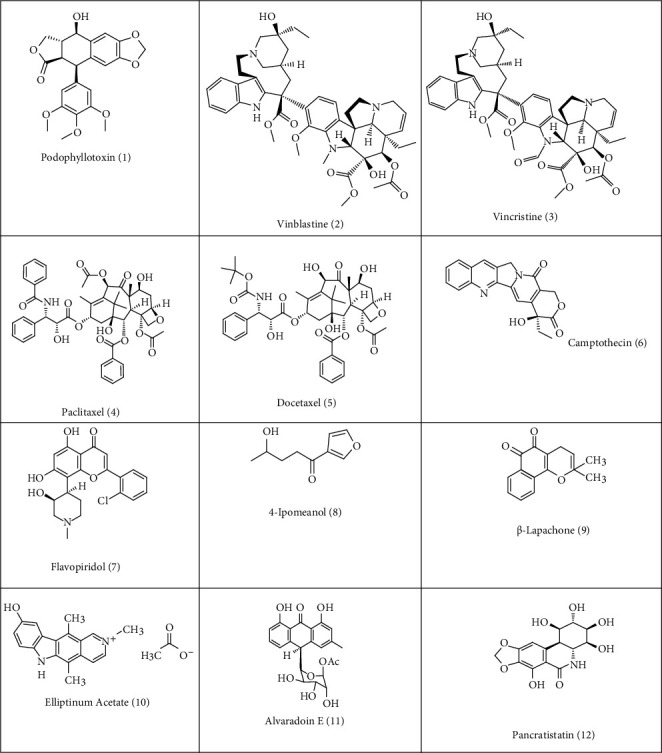
Chemotherapeutic agents obtained from plant resources.

The other antineoplastic agents, 4–ipomeanol (**8**) extracted from *Ipomoeca batatas* (Convolvulaceae) (source sweet potato), have been evaluated [54]. This compound upon metabolic activation by cytochrome P450 (CYPs) enzyme system in the lung cells is converted to DNA binding metabolites. *β*-Lapachone (**9**) inhibits enzyme DNA topoisomerase I and induces delay in the cell cycle at Gap 1 phase or S (synthesis) phase before inducing cell death either through apoptosis or through necrosis in various types of cancers cells of human including breast, ovary, lung, and colon carcinoma cells as shown in Table 3 [78]. Other anticancer agents that are derived from plant and that are under clinical trials include ingenol mebutate, a substance isolated from *Euphorbia peplus*. Elliptinium (**10**) is derivative of ellipticine isolated from *Bleekeria vitensis*. Ten compounds of anthracenone C-glycosides, alvaradoin E-N, were extracted from *Alvaradoa haitiensis* Urb (Picramniaceae) leaves. Collected from Dominican Republic, alvaradoin-E (I) (**11**) was the most active and exhibited significant antileukemic activity at a dose of 0.2 mg/kg per i.p. injection in vivo P388 assay [70]. In *in vivo* hollow fiber experiment, alvaradoin E showed significant inhibition in growth at the i.p. site with KB, LNcaP, and CoI2 cells (Table 3) [69, 79]. Pancratistatin (**12**) is a natural compound initially extracted from spider lily, a Hawaiian native plant of the family Amaryllidaceae, used as anticancer agent (Figure 1).

### 1.2. Anticancer Agent from Marine Natural Products

As mentioned earlier, half of the drugs that are developed or discovered are designed based on the chemical structures from the natural products (NPs) [7]. Most of the therapeutics that are derived are of terrestrial origin. Recently, the focus has been shifted from terrestrial to marine-based products or marine natural products (MNPs) due to novelty in its chemical structure and biological activity [80]. Earth surface is covered by 70% of ocean, which hosts 32 of the 34 phyla discovered on Earth. There is a wide range of diversity among species per square meter in the marine ecosystems. It also has vast diversity among species in each area, and coral reefs show the greatest diversity among all the species. It is not surprising that the ocean is considered as a large unexplored reservoir of diverse and unique NPs [81].

The products obtained from marine environment show significant and higher bioactivity than any other natural products like the one obtained from terrestrial sources. Around 228,500 biologically active compounds have been identified till 2016 [82] from marine source out of which 300 of them have been patented [83]. 37 patents registered in US and Europe are deep sea products. Majority of the novel compounds extracted are from soft-bodied invertebrates residing in the coral reefs. The new generation of pharmaceuticals derived from marine sponge are ready to enter the market [84]. Before 2013, FDA or EMEA approved eight drugs, of which 4 were anticancer drugs. Marine product gained its popularity with the discovery of the cytostatic agent a nucleoside spongouridine (**13a**) and spongothymidine (**13b**) isolated from Caribbean sponge *Tethya crypta.* Based on this, a synthetic refered cytosine arabinoside cytarabine (**14**) was synthesized as an effective antileukemic agent and later FDA approved in the treatment of AME and non-Hodgkin lymphoma (Table 1) [85].

Trabectedin (**15**) was approved by the European union in 2007 to treat reverted incidents of platinum-sensitive ovarian cancer and soft tissue sarcoma and is the first anticancer agent from marine source approved by EU [86]. Trabectedin is derived from ascidian Ecteinascidia turbinate inhabiting Mediterranean and Caribbean Sea. Food and Drug Administration approved it in 2015. The mechanism of action of trabectedin is that it binds to the DNA and prevents the transcription of RNA and inhibits the binding of DNA transcription factor as shown in Table 1. It also controls the microenvironment in and around the tumor by regulating the angiogenic factors and cytokinesis [87, 88].

Synthetic analogue synthesized from the natural marine compound halichondrin (**16**) from sponges such as *Halichondria okadai* and Lyssodendoryx spp. is Eribulin mesylate **(17**) which was approved by FDA in 2010 and by EU in 2011 to treat patients with metastatic breast cancer (Table 1) [89]. Mechanism of action of this synthetic analogue is that it interferes with the dynamic of the microtubule preventing its progression phase and depolymerizing the microtubule and resulting in arrest of the interphase of cell cycle and apoptosis [90].

Another drug that got approval in 2011 by Food and Drug Administration is brentuximab vedotin (**18**). The cytotoxic effect of this drug is due to its combined effect of cytotoxic agent along with tumor-targeted specific monoclonal antibody. It got the approval to treat patients with systemic anaplastic large cell lymphoma and Hodgkin lymphoma. Its cytotoxic effect is due to monomethyl auristatin E (MMAE) and antibody that targets CD30 receptor on the cell membrane found in tumor cell and rarely in normal cells. These two components are linked together by bond [91, 92]. Auristatin is a synthetic version of dolastatin (**19)**, previously obtained from the mollusk *Dolabella auricularia* inhabiting the Indian ocean, and now, it is being extracted from the cyanobacteria *Symploca* mechanism of action (presented in Table 1) which is due to toxic nature; it is not advisable to administer it alone but combination with conjugate antibody specific tumors may be targeted [93]. Brentuximab vedotin interacts with the CD30 receptor on the tumor cell and gets its entry into the cell via clathrin-assisted endocytosis. As it comes in contact with the lysosome, the hydrolytic enzymes in the lysosomes cleaves the bond releasing the MMAE into the cytosol. MMAE prevents the polymerization of the tubulin by binding to it and prevents the progression in to Gap2/M phase of the cell cycle resulting in programmed cell-death [94].

Plitidepsin is a cyclic depsipeptide found in the marine tunicates *Aplidium albicans* in the Mediterranean Sea. Due to its low toxicity and high antitumor activity, FDA in 2006 approved it for the treatment of multiple myeloma (Table 1) [95]. Plitidepsin exerts its anticancer activity by inhibiting the proliferating cell and causes programmed cell death in multiple myeloma (MM), plasmacytoma, prostate cancer pancreatic cancer, and ovarian cancer [39].

Some of the chemicals extracted from the marine natural product with potential chemotherapeutic properties are under clinical trials for their activity shown in Table 2.

Soblidotin is under phase II of the clinical trial. It is extracted from the sea hare *Dolabella auricularia*. It is the synthetic derivative of dolastatin. MOA of soblidotin is that it is a vascular disrupting agent that causes disruption of vascular in the tumor cell. It also has tubulin inhibitory and cytotoxic properties [96].

Elisidepsin is extracted from marine sea slugs *Elysia rufescens*, a marine mollusk. It belongs to the family of Kahalalide compounds and is a mock cyclic depsipeptide [97]. It has shown some promising cytotoxic properties in *in vitro* studies and caused oncolytic cell death rather than apoptosis. It is now under clinical trial for its anticancer properties and for its encouraging therapeutic index [86]. The mechanism by which it exerts its effect is not understood fully, but it has been reported to cause cell death through autophagy via inhibition of Akt/mTOR and stimulation of DAPK (death-associated protein kinase) and independent of caspase-free [98].

Bryostatin 1 is derived from marine bryozoan *Bugula neritina* (Bugulidae). It exhibits both antineoplastic and immuno-potentiation properties.

Other MNPs like coral possess various medicinal properties and are known to have various anticarcinogenic activity [99]. Cytosar-U was the first marine anticancer drug from the coral reefs and is used in treating leukemia and lymphoma. It interferes in the synthesis of DNA in the cells and kills it [100]. There are several literature studies available on the anticancer activity exhibited by corals, and several molecules have also been extracted from various corals [101]. One of the compounds that exhibited anticancer activity by activation of various proapoptotic factors was from the Sinularia species [102]. 5-Episinuleptolide acetate **(20)** (categorized as noncembranoidal diterpene) exhibited significant antiproliferative activity against various cell lines such as K562, Molt 4, and HL 60. In HL60 cancer cell line, there was activation of the downstream apoptotic pathways through Hsp90 inhibition [38]. Sterols present in *Subergorgia reticulate*, a soft coral, induced an apoptotic cycle in the cell that results in the anticancer activity [103]. Secosterol with gorgosterol side chain and with unusual oxygenation pattern on A and B rings was discovered or isolated from soft coral *Lobophytum spp.* [73], and they reported that the discovered compound exhibited antitumor and antileukemic properties against human ovarian tumor and human leukemia cell line. González and his coworkers isolated 13 novel steroids from *Isis hippuris* which is recognized as a species rich source of cytotoxic polyoxygenated steroids [104]. Chemotherapy comes with lots of different side effects, whereas there are no available reports associated with coral reef and side effect [105]. It may be considered as one of the best chemotherapeutic agents with no adverse effects.

Marine blue green algae, also called as Cyanobacteria, are the other areas or fields from which marine drugs can be isolated or extracted. The cell extract of *Calothrix* isolates showed positive results when it was tested against HeLa cancer cell lines. Borophycin (**21**) is a metabolite containing boron, isolated from *Nostoc linckia*, and *N. spongiaeforme* was used against human epidermoid carcinoma and colorectal adenocarcinoma cell line successfully [106, 107]. Later in 1998, Banker and Carmeli described that borophycin can be derived both from terrestrial (*Streptomyces antibioticus*) and marine strains (*S. griseus*) as well. Bryostatins was isolated from the marine organism *Bugula neritina* and was submitted by Jack Rudloe to anticancer drug discovery group at National Cancer institute (NCI). Bryostatins (**22**) are macrolide lactones and are significant inhibitors of protein kinase C [108].

Marine herb is another marine product with some encouraging prospects as an anticancer agent, apart from being rich in vitamins, minerals, proteins, and polysaccharides. It contains a considerable amounts of (2R,3R)-30,40,5,50,7-pentahydroxyflavan-3-yl gallate and 3,4,5-trihydroxybenzoic acid [11]. Different derivatives of marine herbs have been studied extensively for its anticancer activity. Vasanthi and her coworkers carried out a study of the ethanolic extract of *Acanthophora spicifera* against Ehrlich's ascites carcinoma cells in mice and reported from their work that the size of the tumor started to decrease and even the cell count. Other marine herbs that possess cytotoxic activity are *Ulva reticulata* and *Gracileria foliifera* [109]. Fucoidan **(23),** sulfated polysaccharide, was isolated from brown algae. Fucoidan was found to be effective against apoptosis in the human lymphoma HS-Sultan cell line [110, 111]. National Cancer Institute, USA, carried out screening for the cytotoxic activity of the marine herb *Portieria hornemannii* and isolated halomon (**24**)—a pentahalogenated monoterpene—and found out that this biosynthetic product was cytotoxic against cancer cell line [112]. *Sargassum polycystum* is found in South China Sea and is a source of sterols called stigmasterol and *S. carpophyllum* from North China Sea from which different sterols can be extracted which showed significant results when tested on cancer cell lines. These two species are noted for their anticancer activity against various cancer cell line culture [113]. The marine herbs contain high quantity of polyphenols; alkaloids, and polysaccharides; hence, marine herbs are the source of research for many investigators to investigate new entities for pharmaceutical products. Compounds like polyphenols are xenobiotic metabolizing enzymes that can restrict the growth and development of cancer cells, whereas flavonoids can be a potential agent targeted to kill cancer cells or prevent aromatase to prevent the growth of cancer cells [114].

Sea weeds are being a source of a wide variety of chemicals. Several biomolecules with a variety of therapeutic potentials have been isolated such as sulfated polysaccharides, polyphenolic, terpenoids, flavonoids, and lipid-natured secondary metabolites. These secondary metabolites are known to have biological activity. Among them, one of the activities is antimutagenic activity [115]. Brown, red, and green sea weeds have showed significant antiproliferative activity against various malignancy cell-line [116, 117].

Marine microorganism has been the source of anti-infective agents since several years. Alkaloids and quinine isolated from marine microorganisms like bacteria may possess anticancer activity. Adriamycin, daunorubicin, mitomycin C, streptonigrin, and lapachol are analogues of quinine and have shown significant anticancer activity [118].

**Figure 2:**
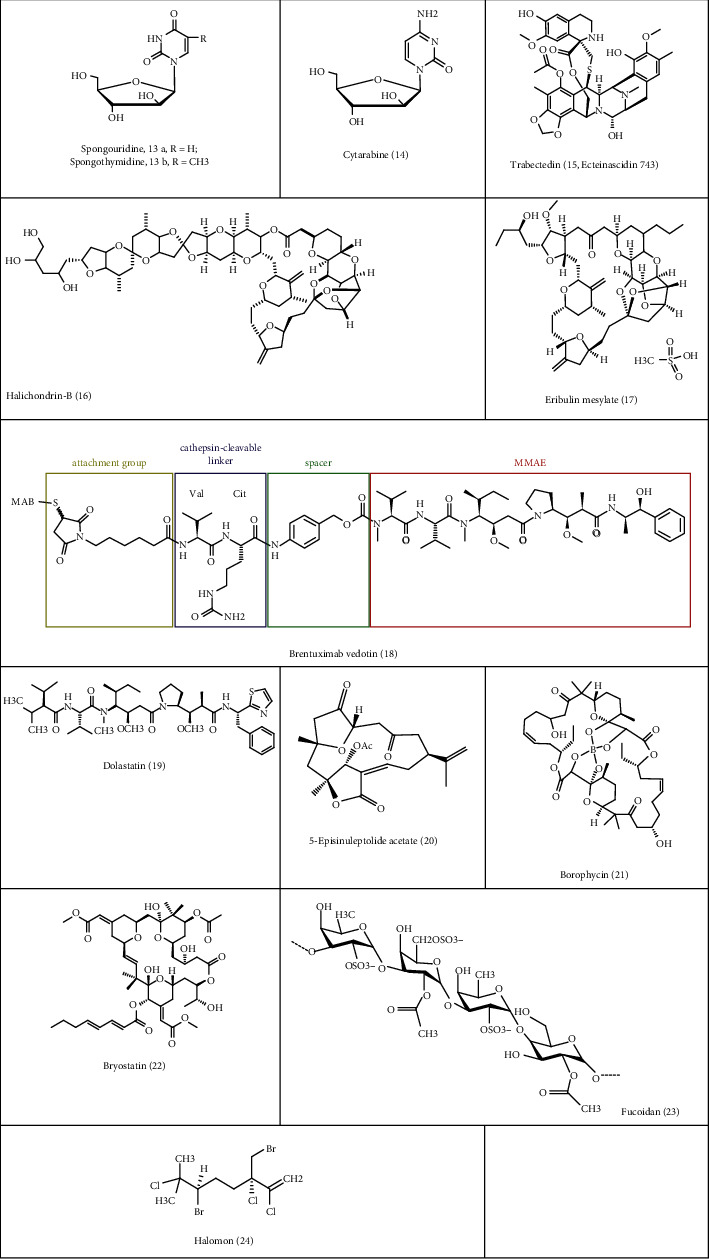
Chemotherapeutic agents obtained from marine resources.

Diatoms are a marine unicellular organism that produces polyunsaturated aldehydes (PUAs). From marine diatoms, three PUAs have been isolated such as *Thalassiosira rotula*, *S. costatum*, and *P. delicatissima*, and these three PUAs (2-trans-4-cis-7-cis-decatrienal, 2-trans-4-trans-7-cis-decatrienal, and 2-trans-4-trans-decadienal) are potential anticancer agents against human colon adenocarcinoma cell line (Table 3) [77] (Figure 2).

### 1.3. Anticancer Agent from Microorganisms

Microorganisms are a source to various structurally different bioactive compounds and have contributed towards large number of antibacterial agents and towards large number of pharmaceutical products and also as nutrients in various food products. Jameel and his colleagues in their review article have reported the beneficial effects of nutraceuticals from bacterial origin that possess various biological activities and anticancer properties [119]. Microorganisms that leave in extreme conditions are a source of chemicals that have significant medical benefits. Microorganisms are the source of secondary metabolites. In recent years, secondary metabolites with antitumor properties from microorganisms have been discovered. There are many bacterial proteins and peptides that possess antiproliferative activity and are currently being used to treat human cancer, and some of them are under clinical trials or explored in *in vitro* studies for their antiproliferative properties. Some of the proteins or peptides that are being used and that exhibit the property are being reported here in this section. The most recently approved anticancer drug derived from the microorganism *Streptomyces hygroscopicus* is everolimus **(25),** which is 40-*O*-(2-hydroxyethyl) derivative of macrolide sirolimus [120]. FDA approved Everolimus in 2011 to treat pancreatic neuroendocrine tumor. It is derived from microorganism *Streptomyces hygroscopicus*. It is an inhibitor of the mammalian target rapamycin (mTOR) (Table 1). Novartis is marketing it under the trade name Afinitor. The FDA in April 2012 approved it for the treatment of renal angiomyolipoma with tuberous sclerosis complex and got approval to treat hormone receptor-positive, HERS2-negative breast cancer in July 2012. The US FDA on 20 July 2012 approved carfilzomib (**26**) (Table 1), for the treatment of relapsed and refractory multiple myeloma in patients who received prior two therapies including bortezomib and immunomodulatory agents. Carfilzomib is a selective protease inhibitor. It is a tetrapeptide-epoxyketone, an analog of epoxomicin (**27**) and isolated from *Actinomycetes* (no. Q996-17) [121]. It is an antitumor agent. Onyx Pharmaceuticals is marketing this product.

Doxorubicin (DOX)—derived from *Streptomyces peucetius* var. caesius—is an antibiotic with antitumor properties. It is an anthracycline compound and is amphiphilic in nature which is due to the aglycone hydrophobic and hydrophilic functional group of amino sugar [122]. The mechanism by which DOX exerts its antitumor activity is by 2 steps. It acts on the nucleic acid of the proliferative cells. First, intercalation of DOX with base pairs on the strands of the DNA results in the inhibition of synthesis of DNA and transcription [123]. Secondly, the iron-free radicals generated cause cellular damage to membranes, protein, and DNA (Table 1) [42]. Doxorubicin is the most common drug used in the chemotherapy. It was approved by the FDA for the treatment of acute lymphoblastic leukemia, acute myeloblastic leukemia, Wilms' tumor, neuroblastoma, soft tissue and bone sarcomas, breast carcinoma, ovarian carcinoma, transitional cell bladder carcinoma, thyroid carcinoma, gastric carcinoma, Hodgkin's disease, malignant lymphoma, and bronchogenic carcinoma. Preet et al. [124] reported that the treatment efficiency increases if doxorubicin was administered along with nisin [124].

Actinomycin D (dactinomycin) is one of the most common antibiotics that are derived from *Actinomyces antibioticus*. With molecular formula C_62_H_86_N_12_O_16_ and mol. wt. of 1.26 kDa, it is effective in the treatment of Wilms cancer, Ewing sarcoma, neuroblastomas, and trophoblastic tumors, primarily in children [125]. It exhibits both antibiotic and antitumor properties. There are various mechanisms through which actinomycin D exerts its cytotoxic and antitumor properties, intercalation to DNA and stabilizes the enzymes topoisomerase I And II [126]. It blocks the expression of DNA and RNA in turn resulting in preventing protein synthesis and inducing programmed cell death (apoptosis) [127].

Bleomycin (BLM) is combination of glycopeptides with antibiotics and cytotoxic properties. BLM is isolated from bacteria *Streptomyces verticillus*. MOA of BLM is it causes the cleavage of DNA as the result of binding of BLM to the DNA and Fe(II). It is used in the treatment of head and neck squamous cell carcinomas, Hodgkin's disease, non-Hodgkin's lymphoma, testicular carcinomas, ovarian cancer, and malignant pleural effusion [128].

Mitomycin C has antitumor properties; it is isolated form actinomyces *Streptomyces caespitosus*. Mechanism of action is it inhibits DNA replication by binding to the DNA during alkylation crosslinking the double strands and preventing the replication of DNA [129]. It is used in the treatment of various cancers such as head and neck, lungs, breast, cervix, bladder, colorectal and anal, hepatic cell carcinoma, and melanoma in addition to stomach and pancreatic cancer.

Other proteins that are extracted from bacteria with activity include bovicin HC5 isolated from *Streptococcus bovis*. It shows structural and functional similarities with other chemical nisin secreted by bacteria [130]. Later, Paiva and his coworkers reported the cytotoxic property of bovicin in their *in vitro* studies carried out against human breast adenocarcinoma (MCF-7) and human liver hepatocellular carcinoma (HepG2) [131].

Laterosporulin 10 (LS10) is defensin-like protein found in *Brevibacillus* sp. With antimicrobial properties, the cytotoxic properties of LS10 were investigated by using normal prostate epithelium cell line (RWPE-1) and five other different human cancer cell lines such as cervical cancer (HeLa), embryonic kidney cancer (HEK293T), fibrosarcoma (HT1080), lung carcinoma (H1299), and breast cancer (MCF-7). It was reported by the investigators that, on all the cell lines, LS10 exhibited dose-dependent cytotoxic property with a maximum concentration of 10 *μ*M on the breast cancer cell line. LS10 was not toxic to normal cells at concentration up to 15 *μ*M. It may be noted that it caused programmed cell death in cancerous cells at lower concentration and necrosis at higher concentration in the cancer cells [132].

Nisin is an antibacterial peptide found in *Lactococcus lactis*. It is a broad-spectrum antibacterial agent which is effective against Gram-positive and Gram-negative bacteria. Joo and his research team studied the anticancer activity and reported that Nisin-A showed antitumor activity against head and neck squamous cell carcinoma (HNSCC) [133].

Lots of effort have been placed over the past few years to explore the anticancer potential of the diverse chemical constituents present in the microorganism as a result of which large number of proteins and enzymes isolated from bacteria with anticancer activity are being studied *in vitro* and can be further explored for their potential in clinical studies in this regard. As clear from the literature, substantial numbers of anticancer agents are from natural sources such as plants, MNPs, and bacteria. Fungi are less explored in this area. Fungi produces large number of secondary metabolites with therapeutical properties. These metabolites have shown tremendous potentials as an antimicrobial agent, with antioxidant activity, and showed encouraging properties as anticancer agents *in vitro*. Jameel and his colleagues reported in their review the biological activity of fungi and its metabolites on the above-said properties [134].

However, it may be noted that, over the past few decades, the fungal metabolites are explored for this purpose, as a result of which there are many fungal-derived metabolite anticancer agents in clinical studies. Fungi produce many novel chemicals or metabolites that possess various biological activities, and a large number of these have been shown to possess cytotoxic activity. Misiek and Hoffmeister in their review article presented the pharmaceutical potentials of chemical constituents of fungal origin with various biological activities such as antiviral and antitumour [135]. Reports have shown that more than 1500 metabolites derived from fungi possess antitumor and antibiotic activity, of which some have entered clinical trial studies and the others serve as the main structures in the synthesis of clinically approved anticancer or antitumor drugs [136]. Among the fungi species, *Aspergillus* and *Penicillium* species contribute the most, around 30% of the isolated metabolite [137].

Fumagillin (**28**) is a fungal metabolite that has been extensively valuated for anticancer activity. It arises from the biosynthesis of sesquiterpenoid (C_15_-nucleus) and polyketides (C_10_ side chain) from the fungus *Aspergillus fumigates* [138]. Based on this, enormous number of semisynthetic analogues of fumagillin are being synthesized to increase its anticancer potential and at the same time to minimize its toxicity. Of these synthesized products, TNP-470 (**29**) and CKD-732 (**30**) were considered as the most potent one. *In vitro and in vivo* studies have shown that TNP-470 inhibits angiogenesis. TNP-470 in 1992 entered clinical trials (Table 2) as an antiangiogenic agent to treat breast, prostrate, brain cancer, and Kaposi sarcoma [139–141]. When compared to TNP-470, CKD-732 (6-O-(4-dimethylaminoethoxy) cinnamoyl fumagillol hemioxalate) was found to be more potent and less toxic, and it entered the clinical trials for its evaluation (Table 2) [56, 59]. In phase I trial, the combination of capecitabine and oxaliplatin with CKD-732 was evaluated for its tolerability, safety, and pharmacokinetics in nine patients suffering from metastatic colorectal cancer and who were progressed to irinotecan chemotherapy. In phase II trial, CKD-732-recommended doses were determined to be 5 mg/m^2^/d, and this recommended dose was combined with other drugs capecitabine and oxaliplatin. Further studies are required on a larger group of population with CKD-732 along with other conventional chemotherapeutic drugs [58].

**Figure 3:**
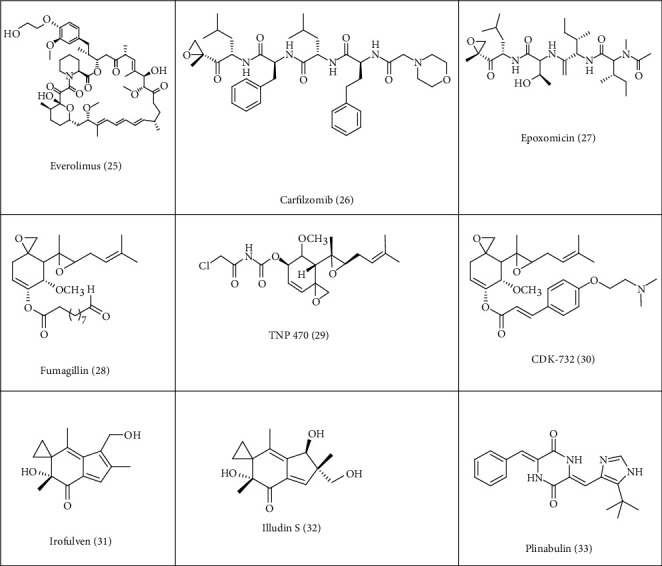
Chemotherapeutic agents obtained from microorganisms.

Irofulven **(31)** is a synthetic analogue of sesquiterpene illudin **(32)**. It has a significantly low therapeutic index and is more selective towards human tumor cells [142]. It is a DNA alkylating agent (Table 2). The US FDA in 2001 gave approval for clinical trials. MGI Pharma and National Cancer Institute conducted the clinical trials. Irofulven showed encouraging results in the trials. The malignant tumors shrinked, and even the tumors that were resistant to the drugs also decreased in size. [61]. It showed promising outcomes in pancreatic cancer patients who had ceased responding to treatment. A phase III clinical investigation was initiated as a result of these positive outcomes [60]. Plinabulin (**33**) is an orally active diketopiperazine derivative with potential antineoplastic activity. Plinabulin selectively targets and binds to the colchicine-binding site of tubulin, thereby interrupting equilibrium of microtubule dynamics (Figure 3).

### 1.4. Anticancer Agents from Endophytes

Endophytes are the organisms such as bacteria, yeast, and fungi that live in the plant or that spend some part of their life cycle inside the plant without causing any pathogenic effect [143]. There has been an increase in the evidence that suggest that the association between plants and microorganism such as endophytes, fungi and rhizosphere bacteria contain large number of untapped chemicals that need to be explored for their pharmaceutical potentials. These NPs from microorganism contain diverse bioactive chemicals. The chemical structure and the biological activity of the compound isolated from the plant associated with microbial strain has been reported in the review article [144].

There are around one million fungal endophytes [63]. Taxol was discovered in 1993 to be produced by the fungus that lives in the yew tree. It has now been isolated from a large variety of endophytic fungi, allowing it to be manufactured by growing these kinds of fungi, which will lower the time and cost of manufacturing [145, 146]. Paclitaxel, on the other hand, is the first cytotoxic secondary metabolite of fungal origin to be used in clinical practice which is discussed above.

**Figure 4:**
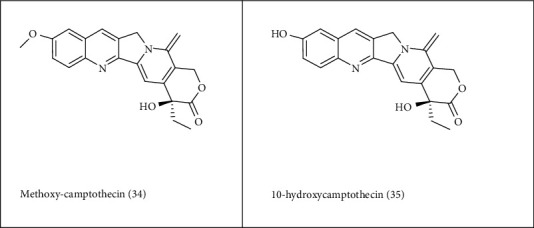
Chemotherapeutic agents obtained from endophytes.

It was first reported that the endophytic fungi *Entrophospora infrequens* from *Nothapodytes foetida* have the ability to produce camptothecin (CPT) methoxy-camptothecin (**34**) and 10-hydroxycamptothecin (**35**), and these two compounds are the analogues of CPT with anticancer potentials and low toxicity [147]. Another endophytic fungus that produces CPT is a partially identified phycomycete fungus (RJMEF001) found in the bark of *N. foetida* in India [148]. *Alternaria* sp. and *Fusarium oxysporum* were isolated from the phloem of *C. roseus* and were responsible for the production of vinca alkaloids [149]. It has been reported that other compounds like aryl tetralignan podophyllotoxin is produced by the endophyte *Phialocephela fortinii* that was isolated from the host plant *Podophyllum peltatum* rhizome [150] (Figure 4).

## 2. Conclusion

The primary goal of research and development of NPs is to develop a therapeutic agent to treat human disease as these compounds derived from various natural resources are safer with lesser side effects and are of low cost. Natural source serves as a source of anticancer agents. In the present review, anticancer agents from a natural source have been reviewed. As reported, a large number of phytochemicals that are derived from various natural sources are under various stages of clinical developments, which shows that the natural source is still a practical way. The Earth holds a large amount of large diverse biological source, and only a few fractions of it have been explored. It continues to provide a large variety of diverse biologically active compounds that can be exploited for the development of new novel and clinically improved agents to treat human cancer with less suffering can be achieved. Most new chemicals that are being discovered are from the terrestrial origin, and some of these compounds are already in clinical use. Although marine sources show vast diversity among species and diversity among the compounds, it has been less explored due to the scarcity of technology in exploring the extreme environmental conditions, culturing the sample, and identification of the lead compound. To develop a drug with clinical application, a chemical must be identified. The development of innovative technologies has solved this problem. The advances in technologies like sample strategy and nanoscale NMR technologies to illustrate the chemical structure and biotechnology have played a vital role in the development of an ideal drug of marine origin. As a result of this, many of the chemotherapeutic agents from marine origin have entered the market for clinical use. Research on plant endophytes has revealed that it produces bioactive compounds that were originally taught to be produced by the plant in which it resides. Endophytes are known to produce superior compounds than their hosts. These endophytes may be exploited for their bioactive compounds by culturing it in the laboratory and isolating the key compounds that may be an ideal anticancer agent and lead compound of plant origin. The isolated compound may be used in improving the existing anticancer drugs or develop a structurally superior therapeutic agent with fewer side effects. It may be noted that the research on NPs is basically restricted to the academician. We need an effective collaboration between academics and pharmaceutical companies, and with the help of advanced technology, a new potential, novel anticancer drug that is safer, with less side effects and of low cost, can be produced.

## Figures and Tables

**Table 1:** Anticancer agent from natural source that has been already used in the treatment of various cancers in humans and FDA-approved agents.

Name	Source	Name of the source	MOA	Target and type of cancer	Reference
Vincristine vinca alkaloid	Plant	*Catharanthus roseus*	Inhibits self assembly of microtubule and induces assembly of tubulin	Children's leukemia	[22, 23]
Vinblastine	Plant	*Catharanthus roseus*	Binds to microtubule proteins in the mitotic spindle and prevents cell division during metaphase	Hodgkin's disease (lymphoid cancer)	
Etoposide	Plant	*Podophyllum peltatum*	Inhibits enzyme topoisomerase II in DNA replication	Testicular cancer (along bleomycin and cisplatin)	[24]
Camptothecin quinoline alkaloid	Plant	*Camptotheca acuminata*	Topoisomerase I	Irinotecan and topotecan ovarian and colorectal cancer	[25]
Paclitaxel	Plant	*Taxus brevifolia*	Stabilizes microtubules and leads to mitotic arrest	Ovarian, breast, and other tumors in clinic	[26, 27]
Docetaxel	Plant		Antineoplastic activity: I—inhibition of microtubular depolymerization II—attenuation of the effects of Bcl-2 and Bcl-xL gene expression		
Taxane	Plant		Disruption of microtubule function	Early and metastatic breast cancer	[28]
Homoharringtonine	Plant	*Cephalotaxus harringtonii*	Inhibition of protein synthesis in the ribosome of cancer cells	Chronic myeloid leukemia after failure of 2 or more tyrosine kinase inhibitors	[29]
Ingenol mebutate	Plant	*Euphorbia peplus*	Protein kinase C (PKC) activator	Antileukemic	[30]
Cytarabine	Marine	*Sponge*	Sugar moiety of cytarabine hinder the rotation of the molecule within the DNA. Stops replication of DNA in the S phase of cell cycle.	AME and non-Hodgkin lymphoma FDA approved	[31, 32]
Ecteinascidin-743 (synthetic compound like sponge)	Marine	*Ecteinascidia turbinata*	Interacts with the minor groove of DNA and alkylates guanine at the N2 position	Metastatic breast cancer FDA approved 2010	[33, 34]
Dolastatin-10, (MMAE-synthetic)	Marine	*Dolabella auricularia*	Binds to the microtubule and prevents polymerization inhibiting the G/M phase cell cycle and apoptosis	Hodgkin's lymphoma, systemic anaplastic large cell lymphoma, cutaneous, peripheral T-cell lymphomas FDA approved	[35]
Eribulin	Marine	*Marine sponge*	Inhibition of microtubule polymerization—by binding to high-affinity sites on *β*-tubulin at the exposed (plus) ends of growing microtubules	Unresectable and metastatic liposarcoma FDA approved	[36]
Eribulin mesylate	Marine	*Marine sponge,* Halichondria okadai	Inhibition of the growth phase of the microtubule	Metastatic breast cancer approved	[36]
Cytosar-U	Marine	*Coral reef*	Interferes in DNA synthesis in cells and kills it	Leukemia and lymphoma	[37]
5-Episinuleptolide acetate	Marine	Coral reef	This diterpenoid led to caspases-3, -8, and -9 activation as well as PARP cleavage. It also induced ROS generation, calcium accumulation, and disruption of mitochondrial membrane potential.	Leukemia	[38]
Plitidepsin	Marine tunicates	*Aplidium albicans*	Inhibits proliferating cell and causes programmed cell death	Multiple myeloma FDA approved 2006	[39]
40-O-(2-hydroxyethyl-rapamycin	Microorganism	*Streptomyces hygroscopicus*	Protein kinase inhibitor and cytochrome P450 3A4 inhibitor; modulates human dendritic cell function	Pancreatic neuroendocrine tumors: FDA 2011, renal angiomyoloma with tubererous sclerosis FDA April 2012, hormone receptor-positive, HERS2-negative breast cancer FDA July 2012	[40]
Carfilzomib	Microorganism	*Actinomycetes* No. Q996-17	Selective protease inhibitor	Relapsed and refractory multiple myeloma—FDA July 2012	[41]
Doxorubicin (DOX)	Microorganism	*Streptomyces peucetius* var. caesius	Intercalation of DOX-base pairs on the strands of the DNA, resulting in the inhibition of synthesis of DNA and transcription, the iron free radicals generated causes cellular damage to membranes, protein, and DNA	Acute lymphoblastic leukemia, acute myeloblastic leukemia, Wilms' tumor, neuroblastoma, soft tissue and bone sarcomas, breast carcinoma, ovarian carcinoma, transitional cell bladder carcinoma, thyroid carcinoma, gastric carcinoma, Hodgkin's disease, malignant lymphoma, and bronchogenic carcinoma 1974	[42]

**Table 2:** Anticancer agent from natural sources that are under various stages of Clinical trials.

Name of compound	Source	Name of the source	MOA	Clinical trials against cancer	Reference
Flavopiridol	Plant	*Amoora rohituka, Dysoxylum binectaiferum*	Blocking the progression at gap 1 (G1) or gap2 (G2) phase of the cell cycle	Phase I clinical trial: dose-dependent toxicity Phase II clinical trial patients: colorectal cancer, prostate cancer, renal cell carcinoma, small cell lung carcinoma, non-lymphoma, Hodgkin's, and chronic lymphocytic leukemia	[52, 53]

4-Ipomeanol	Plant	*Ipomoeca batatas*	Inhibits DNA topoisomerase I and induces delay in the cell cycle at gap G-1 phase or S phase	Clinical trial: lung cancer-specific antineoplastic agent	[54]

Ingenol mebutate	Plant	*Euphorbia peplus*	—	Under clinical trial Discontinued production due to increase risk of nonmelanoma skin cancer	[30]

Elliptinium acetate: a derivative of ellipticine	Plant	*Bleekeria vitensis*	Topoisomerase II inhibitor and intercalating agent, inhibiting DNA replication and RNA and protein synthesis	Under clinical trial	[55]

TNP-470: an analog of fumagillin	Fungi	*Aspergillus fumigates*	Inhibits angiogenesis	Clinical trials for its antiangiogenic agent to treat breast, prostrate, brain cancer, and Kaposi sarcoma	

CKD-732 (O-(4-dimethylaminoethoxycinnamoyl)fumagillol)	Fungi	*Aspergillus fumigates*	Inhibits angiogenesis	Phase I: in combination was checked for tolerability and safety Phase II: recommended dose determined	[56–59]

Irofulven synthetic analogue of illudin S	Fungi	*Omphalotus illudens*	Alkylating agent	Irofulvene in treating patients with stage III or stage IV pancreatic cancer. Clinical trial was conducted. Details not available.	[60, 61]

Plinabulin	Marine fungus	*Aspergillus ustus*		Clinical trial: positive result	[62, 63]

**Table 3:** Anticancer agent that shows chemotherapeutic properties on various cell lines.

Name of the compound	Source	Name of the source	Mechanism of action	Activity against cell line	Reference
4-Ipomeanol	Plant	*Ipomoeca batatas*	Inhibits DNA topoisomerase I and induces delay in the cell cycle at gap G-1 phase or S phase	Induces cell death in human carcinoma including breast, ovary, lung, and colon carcinoma cells	[54, 68]

Alvaradoin E (**10**)	Plant	*Alvaradoa haitiensis* Urb	—	Antileukemic activity KB, LNcaP, and CoI2 cells	[69, 70]

Laulimalide and Isolanulide	Marine	*Hyatella sp*	Microtubule—stabilizing agent—inhibited the P-glycoprotein responsible for multiple drug resistance in tumor cells	Cytotoxic against KB cell line	[71]

5-Episinuleptolide acetate	Marine	*Sinularia* sp	—	Cytotoxic against cell lines like K562, Molt 4, and HL 60.	[38]

Secosterol	Marine	*Lobophytum sp*	—	Antitumor and antileukemic against human ovarian tumor and human leukemia cell lines	[72, 73]

Fucoidan-sulfated polysaccharide	Marine	Brown algae	Activation of the host immune responses	Effective against apoptosis, human lymphoma, and HS-Sultan cell line	[74]

Halomon pentahalogenated	Marine	*Portieria hornemannii*	Acts as demethylating agent	Cytotoxic cancer cell line	[75, 76]

Polyunsaturated aldehydes (PUAs)	Marine diatoms	*Thalassiosira rotula*, S. *costatum*, and *P. delicatissima*	—	Anticancer against human colon adenocarcinoma cell line	[77]

## Data Availability

The data used to support the findings of this study are available within the article.
